# Hypotussic cough in persons with dysphagia: biobehavioral interventions and pathways to clinical implementation

**DOI:** 10.3389/fresc.2024.1394110

**Published:** 2024-06-12

**Authors:** Justine Dallal-York, Michelle S. Troche

**Affiliations:** Laboratory for the Study of Upper Airway Dysfunction, Department of Biobehavioral Sciences, Teachers College, Columbia University, New York, NY, United States

**Keywords:** cough, dysphagia, aspiration, swallowing, airway, hypotussia, dystussia, treatment

## Abstract

Cough is a powerful, protective expulsive behavior that assists in maintaining respiratory health by clearing foreign material, pathogens, and mucus from the airways. Therefore, cough is critical to survival in both health and disease. Importantly, cough protects the airways and lungs from both antegrade (e.g., food, liquid, saliva) and retrograde (e.g., bile, gastric acid) aspirate contents. Aspiration is often the result of impaired swallowing (dysphagia), which allows oral and/or gastric contents to enter the lung, especially in individuals who also have cough dysfunction (dystussia). Cough hyposensitivity, downregulation, or desensitization- collectively referred to as *hypotussia*- is common in individuals with dysphagia, and increases the likelihood that aspirated material will reach the lung. The consequence of hypotussia with reduced airway clearance can include respiratory tract infection, chronic inflammation, and long-term damage to the lung parenchyma. Despite the clear implications for health, the problem of managing hypotussia in individuals with dysphagia is frequently overlooked. Here, we provide an overview of the current interventions and treatment approaches for hypotussic cough. We synthesize the available literature to summarize research findings that advance our understanding of these interventions, as well as current gaps in knowledge. Further, we highlight pragmatic resources to increase awareness of hypotussic cough interventions and provide support for the clinical implementation of evidence-based treatments. In culmination, we discuss potential innovations and future directions for hypotussic cough research.

## 1 Introduction

**Figure 1:**
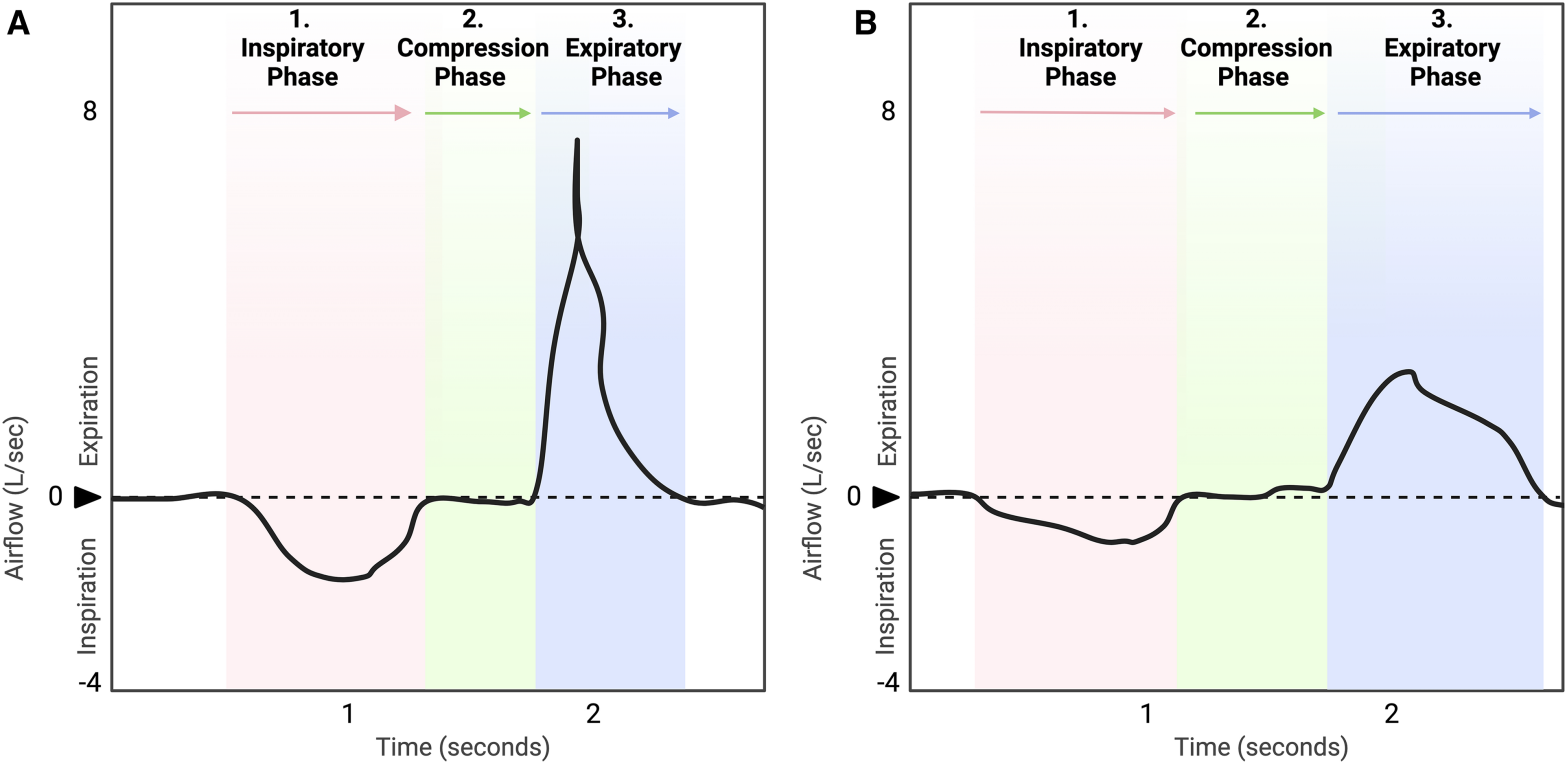
Healthy, typical cough waveform (**A**) is compared to hypotussic cough waveform (**B**). A timely and coordinated cough rapidly reconfigures the ventilatory pattern to produce a three-phase airflow sequence (**A**). In eutussia (normal cough), (8, 9) this includes a period of initial inspiration, followed by laryngeal compression, and ballistic air expulsion during expiration (5, 7, 10, 20). In hypotussia (**B**), changes in slope and duration during inspiratory and expiratory phases with compression phase leak contribute to poor cough outcome metrics and manifest clinically as reduced cough effectiveness.

Cough is a vital, life-sustaining behavior essential for pulmonary homeostasis (1–4). The major function of cough is to generate the high velocity airflows and shearing forces required to expel unwanted material from the airways and allow breathing to occur unobstructed (1, 5–7). Unfortunately, disordered cough (or dystussia, Table 1) is quite prevalent, especially in the context of dysphagia or swallowing disorders, exacerbating the risk of the deleterious effects which can be associated with uncompensated aspiration (3, 5, 18, 19). Hypotussia, or downregulated cough, manifests as too little or insufficient coughing (2, 8, 15). It is primarily associated with cough hyposensitivity and/or reduced cough effectiveness (2, 8–11). The reduced cough effectiveness observed in hypotussia (12) is characterized by distinct impairments in cough motor airflow patterns [Figure 1, Supplementary File S1: Lowell et al. (2), McGarvey et al. (12)]. One of the main clinical outcome measures and indicators of uncompensated aspiration is peak cough flow or peak expiratory flow rate (hereafter, referred to as PEFR) (21–23). Together, sensorimotor impairments in cough can result in uncompensated aspiration over time, which can be life-threatening (18, 19, 24).

**Table 1:** Description of clinical presentations of cough.

Key term	Descriptions
Eutussia	Normal cough (8, 9)
Dystussia	Pathological or impaired cough function; disordered cough; can also include the spectrum of alterations in cough sensation spanning from hyper- to hyposensitive cough (2, 9, 10)
Hypertussia	Upregulated, sensitized cough; refers to syndromes where too much coughing is produced; cough hypersensitivity (5, 9, 11–14)
Hypotussia	Downregulated, desensitized cough; refers to syndromes where too little coughing occurs; cough hyposensitivity (2, 8, 9, 15, 16)
Atussia	Absence of cough (8, 9, 17)

**Figure 2:**
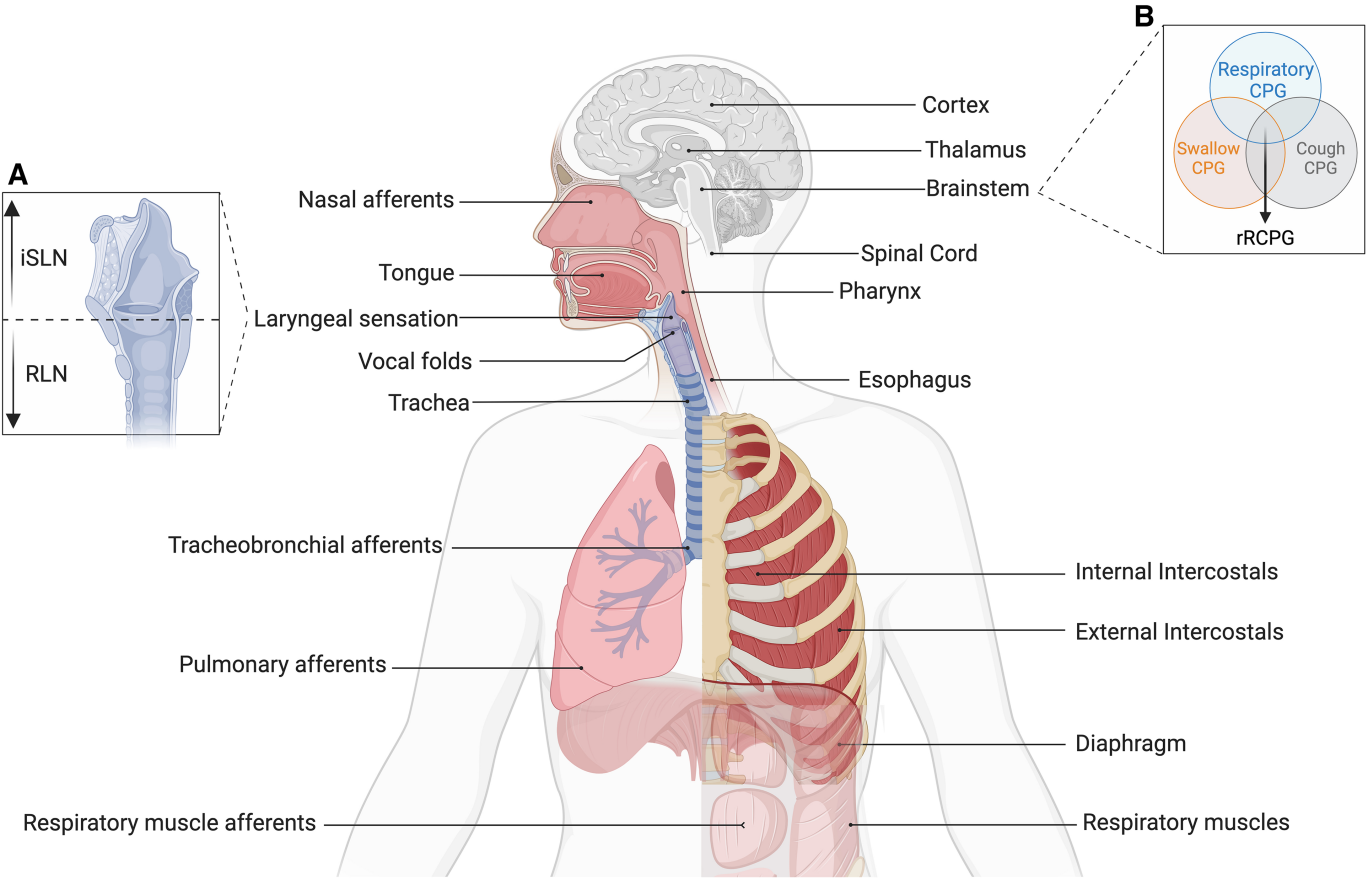
Pathophysiology of hypotussia in individuals with dysphagia. Across the neural axis, changes in sensation, motor control or sensorimotor integration involved in cough may contribute to hypotussia (12). Structural changes may limit one's ability to coordinate breathing and swallowing, or generate adequate pressure for high velocity airflows during cough. These may include: (1) altered compliance of the chest wall, (2) weak/spastic inspiratory (e.g., diaphragm, external intercostals) and expiratory (e.g., abdominals, obliques, internal intercostals) muscles, (3) vocal fold and upper airway pathologies (6, 10, 12, 18, 19). Disruptions in neural signaling of sensorimotor pathways may also contribute to hypotussia, including dysfunctional signal reception, transmission, processing, and/or output in one, or several neural substrates: (1) pulmonary, tracheobronchial, and laryngeal receptors that receive cough stimuli input (10, 12, 25–27), (2) vagal afferents of the airways that transmit sensory input to the central nervous system, including internal superior (iSLN) and recurrent laryngeal nerves (RLN) [Box **A**] (10, 25, 26), (3) central pattern generators (CPG) for swallow, cough, and breathing integrate sensory input to generate a reconfigured respiratory CPG (rRCPG) to execute cough [Box **B**] (4, 28), and (4) subcortex and cortical structures involved with filtering, perceiving, and processing discriminative and affective characteristics of the sensory stimuli, leading to execution of volitional cough, or suppression/augmentation of reflexive cough (4, 10, 12, 13, 25–27, 29). In summary, alterations to composite anatomy, musculature, or neural pathways involved in swallow, cough, and/or breathing may also result in hypotussia, compounding the impact of aspiration. iSLN, internal branch of the superior laryngeal nerve; RLN, recurrent laryngeal nerves; CPG, central pattern generator; rRCPG, reconfigured respiratory central patter generator.

Hypotussia may occur due to underlying afferent and efferent deficits in the neural control of cough [Figure 2. Pathophysiology of hypotussia, please see Supplementary File S1: Lowell et al. (2), for a detailed neurological review]. In individuals with dysphagia, hypotussia is concerning as it results in an inability to adequately sense or move aspirate material from the airways (2, 15, 16). This can have a significant impact on quality of life and patient health (30–32). Chronic aspiration due to concomitant dysphagia and hypotussia increases the risk for fatal respiratory infections (3, 33). Further, dysphagia-related hypotussia is associated with increased rates of pneumonia, reintubation, hospital readmission, and costs of care (31, 34–38). Complex deficits across the continuum of airway protection (i.e., swallow to cough) co-occur in multiple patient populations including: Parkinson's disease (39, 40), amyotrophic lateral sclerosis [ALS; (23, 41–43)], multiple sclerosis (44), atypical parkinsonism (31), progressive supranuclear palsy [PSP, (45)], cerebellar ataxia (25), stroke (41, 46), head and neck cancer (47–49), cerebral palsy (50), spinal cord injury (51), lung transplantation (52), and cardiac surgery (37). Thus, to improve health and quality of life for this large and heterogeneous group of patients, it is critical that rehabilitation efforts include cough (4, 15, 31, 38). These interventions should address the need for an immediate cough in response to aspiration, but also the need for cough to clear the lower airways and address the long-term impacts of uncompensated aspiration.

There is rising recognition that speech-language pathologists (SLPs) must be adept in treating the multiple behaviors of airway protection, including both swallow and cough (15). In a recent survey, 85% of SLPs reported not only that they evaluate cough, but feel that the results from cough assessment should influence clinical practice patterns (53). Moreover, although cough is within the SLPs scope of practice for provision of dysphagia services (54, 55), only 33% of clinicians report receiving formal training on cough (53). Limited visibility, time and resources on viable treatments for cough disorders are also barriers to clinical uptake (10, 56). The goal of this manuscript is to provide clinicians working with dysphagia populations with a pragmatic, clinically relevant resource to address this unmet didactic need. To achieve this goal, we will provide an overview of the literature related to hypotussic cough behavioral interventions, including both exercise-based rehabilitative approaches and compensations. Additionally, we will provide resources to facilitate the implementation of these approaches in the care of people with airway protective dysfunction.

## 2 Hypotussic cough interventions

**Figure 3:**
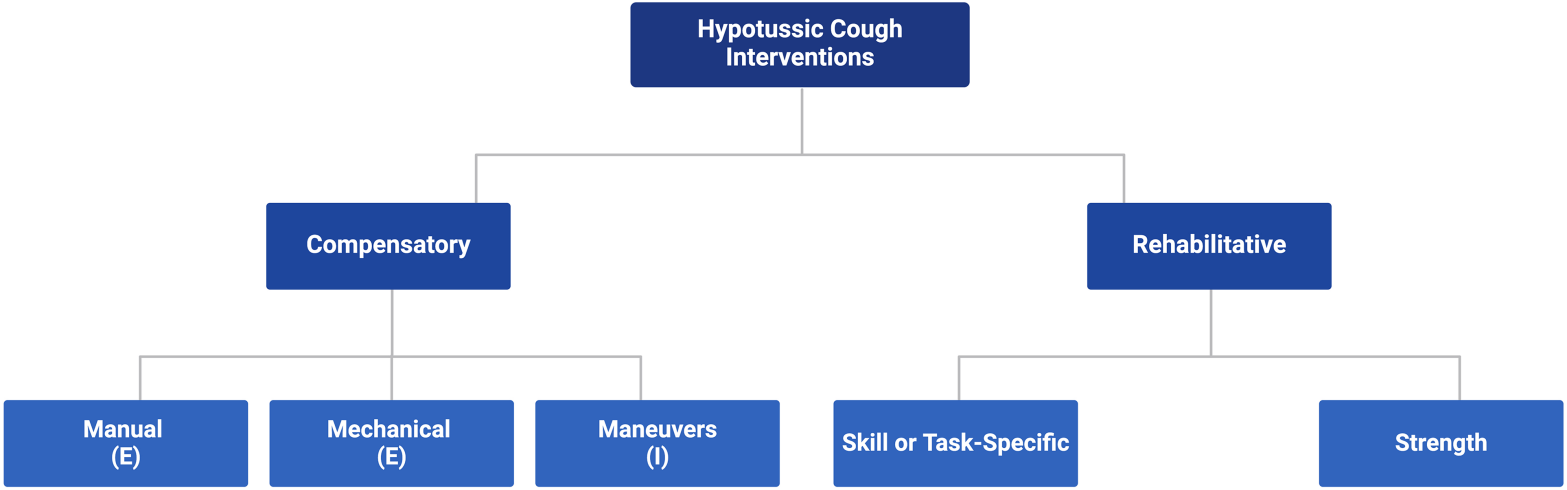
A framework for treatment of hypotussic cough. E, external; I, internal.

Using clear, familiar terminology, we define and structure hypotussic cough interventions into a comprehensive framework (Figure 3) to help scaffold current evidence-based treatment options for patients and members of their care team. Guided by the existing literature [Supplementary File S1: Chatwin et al. (57), Huckabee et al. (58), Lee et al. (5), Zimmerman et al. (59)], we categorize cough behavioral interventions into two domains, compensatory and rehabilitative, along with their respective subcategories.

### 2.1 Domains of hypotussic cough interventions: rehabilitative vs. compensatory

Hypotussic cough interventions fall into two key domains, approaches that are rehabilitative vs. those that are compensatory (Figure 3). Rehabilitative interventions provide long-term improvements to physiologic and functional cough outcomes and are hypothesized to directly or indirectly alter the underlying neural substrates involved in cough (58–60). These interventions are implemented to target the underlying physiology in cough impairments, and seek to restore function with sustained improvements that extend beyond their immediate application or use within treatment (58–60). In contrast, compensatory interventions are primarily strategies which provide short-term or transient enhancements to physiologic and functional airway protection outcomes (58–60). Compensatory strategies create immediate adjustments to cough function which may improve airway clearance and aspiration management while in use, but do not necessarily alter cough physiology and likely do not leave enduring changes that extend beyond the treatment period (58–60). Depending on the underlying pathophysiology of hypotussic cough impairments, cognitive status, patient preferences and goals of care, rehabilitative and compensatory strategies may be used independently or in combination.

### 2.2 Rehabilitative interventions: strength vs. skill/task-specific paradigms

Here we categorize rehabilitation paradigms for hypotussia as either strength or skill/task-specific interventions. Strength based rehabilitation aims to increase force generating capacity of peripheral muscles through targeted exercise programs (7). These programs are typically prescribed for airway protection impairments that are due to underlying peripheral muscle weakness and are heavily dependent on sports medicine principles (61, 62) such as progressive overload, intensity, frequency of exercise, and specificity of movement type (59–61, 63, 64). In turn, it is hypothesized that peripheral neuromuscular adaptations including motor unit recruitment, muscle fiber type shift, and myofiber hypertrophy may be observed (60, 61, 64–66). In contrast, skill-based or task-specific rehabilitation aims to increase the coordination and sequence involved in the central mechanisms that execute airway protective behaviors (58, 59, 67, 68). These programs place a unique emphasis on the critical role of motor control to promote increased cognitive awareness of coordinated, skilled tasks (58, 59, 67, 68). Task-specific treatments are largely driven by principles of motor learning including specificity of practice, task challenge, and impact of biofeedback with the goal of promoting functional reorganization and change in higher level cortical networks (58, 59, 69). Excellent reviews from the fields of dysphagia and respiratory physiology further elucidate the differences between these treatment approaches and/or expand on underlying mechanisms of change (please see Supplementary File S1: Reading List).

### 2.3 Strength based rehabilitation of cough

Respiratory strength training (RST) is a behavioral intervention applied in patient populations with breathing impairments. Given that inspiratory and expiratory muscles are necessary to produce an effective cough and maintain airway patency there is a strong physiologic rationale for targeting both inspiratory (maximum inspiratory pressure) and expiratory (maximum expiratory pressure) force-generating capacity to address hypotussia and improve cough strength and airway clearance in individuals with dysphagia (2, 62). As there is a growing and robust literature set on RST in airway protection, the studies reviewed in this section will focus on randomized controlled trials, quasi-experimental investigations, and cohort studies which directly report on objective cough outcome measures. Studies are further limited to investigations in adults. Case studies are not included.

The primary goal of RST is to increase inspiratory (inspiratory muscle strength training, IMST) and expiratory (expiratory muscle strength training, EMST) muscle strength to preserve or improve outcomes such as ventilation, cough effectiveness, and airway safety (35, 70–73). Overall, the benefits of RST have been highlighted in multiple patient populations including: Parkinson's disease (38, 71, 73–75), ALS (73, 76, 77), multiple sclerosis (44), Huntington's disease (78), sedentary elderly (79), chronic obstructive pulmonary disease (80), failure to wean and/or ventilator dependence (81), stroke (70, 82), head and neck cancer (83) and cardiac surgery (35). Across these patient populations, descriptive data with information on respiratory load, number of repetitions, frequency and duration of treatment paradigms are depicted in Table 2.

**Table 2:** Respiratory strength training paradigms and cough outcomes.

Patient population	Respiratory load (%MIP/MEP)		Repetitions (#reps/#sets/session)	Frequency (days/week)	Duration (weeks)	Cough type		Reference
	% MIP	% MEP				Voluntary cough	Reflex cough	
Multiple sclerosis	–	40%–80%	6/4/1	5/7	8	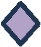	–	Chiara et al. (44)
Parkinson's disease	50%–75%	–	5/5/1	6/7	8	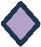	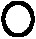	Reyes et al. (74)
	–	50%–75%	5/5/1	6/7	8	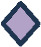	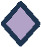	
	–	75%	5/5/1	5/7	4	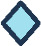	–	Pitts et al. (71)
	–	50%–75%	5/5/1	6/7	8	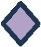	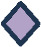	Reyes et al. (73)
	–	75%	5/5/1	5/7	4	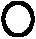	–	Sapienza, et al. (84)
	–	75%	5/5/1	5/7	4	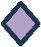	–	Sevitz et al. (85)
	–	75%	5/5/1	5/7	4	–	–	Troche et al. (75)
	–	75%	5/5/1	5/7	5	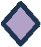	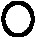	Troche et al. (38)
Huntington's disease	30%–75%	30%–75%	5/5/1	6/7	16	–	–	Reyes et al. (78)
Cardiovascular surgery	50%	50%	5/5/1	5/7	3–10	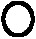	–	Donohue et al. (35)
Acute stroke	50%	50%	10/5/1	7/7	4	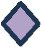	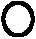	Kulnik et al. (82)
Unilateral stroke	30%–60%	15%–75%	5/5–6/1–2	5/7	6	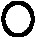	–	Liaw et al. (86)
Ischemic stroke	–	60%	5/5/1	5/7	5	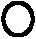	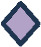	Hegland et al. (70)
Head and neck cancer	–	75%	5/5/1	5/7	8	–	–	Hutcheson et al. (49, 83)
Partial laryngectomy	–	Mean of 3x	5/5/1	5/7	4	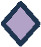	–	Palmer et al. (87)
Total laryngectomy	–	80%	5/5/1	5/7	4	–	–	Van Sluis et al. (88)
Amyotrophic lateral sclerosis	30%	30%	5/5/1	5/7	12	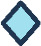	–	Plowman et al. (76)
	–	50%	5/5/1	5/7	8	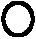	–	Plowman et al. (72)
	–	50%	5/5/1	5/7	5	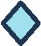	–	Plowman et al. (23, 77)
Multiple system atrophy	–	75%	5/5/1	5/7	8	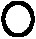	–	Srp et al. (89)

Dash = Not assessed in investigation; MIP, maximum inspiratory pressure; MEP, maximum expiratory pressure.

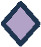
 PEFR Improved.

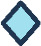
 Other objective cough airflow metric improved.

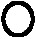
 Tested but no statistically significant difference.

In light of the important relationship between metrics of expiratory muscle strength, namely maximum expiratory pressures (MEPs) and cough airflow (90), EMST has garnered significant research and clinical interest as an effective therapeutic strategy to improve cough function. EMST is an active rehabilitation approach which uses a calibrated one-way, spring-loaded pressure threshold device to overload and mechanically drive the expiratory muscles. Typically, EMST regimens involve breathing through the device for 25 repetitions (five sets of five repetitions) five days per week for four to five weeks (35, 62, 70, 72, 75, 78, 81). Although EMST has consistently been shown to improve MEPs, there is a rising number of investigations which demonstrate its efficacy to improve measures of voluntary and reflex cough (70, 71, 74, 82–88).

EMST has been found to improve voluntary and reflex cough in multiple populations. In one of the first studies of EMST for cough in patient populations, ten male participants diagnosed with Parkinson's disease completed a 4-week EMST program, with improved cough effectiveness, evidenced by changes in various measures of cough airflow (71). A significant decrease in compression phase duration and expiratory phase rise time was observed after EMST, leading to a significant increase in cough volume acceleration (66) during voluntary cough testing. Similar results were found in sedentary older adults (79), where following 4-weeks of EMST, reflex cough demonstrated a 61% increase, rising significantly from 4.98 ± 2.18 to 8.00 ± 3.06 L/s (F1,17 = 29.62, *p* < 0.001). Further, in an investigation which compared respiratory and cough-related parameters between individuals with multiple sclerosis and healthy controls, results from eight weeks of EMST revealed statistically significant increases in MEP and PEFR (44). Moreover, Hegland and colleagues (70) found that measures of reflex, but not voluntary, cough airflow improved after five weeks of EMST in individuals with a history of ischemic stroke.

Some of the most promising investigations on EMST which have shown improvements in cough outcomes have been conducted in neurodegenerative disease. In Parkinson's disease, four and five weeks of EMST have been shown to improve various metrics of cough function (38, 71, 84, 85). In a recent randomized controlled trial in Parkinson's disease, 5-weeks of EMST resulted in improvements in MEPs by 22 cmH2O, (*p* < 0.001, *d* = 0.53) and/or voluntary cough strength by 0.17 L/s (*p* < 0.001, *d* = 0.06) (38). Moreover, Reyes and colleagues (74) found that two months of home-training sessions of EMST in individuals with Parkinson's disease resulted in a significant effect on MEP (*d* = 1.40) and voluntary PEFR (*d* = 0.89) when compared to control and IMST groups. In later works by this group, similar findings were observed with an additional benefit of adding air stacking to EMST training to improve both reflex and voluntary PEFR as compared to EMST alone (73). Interestingly, in individuals with ALS, a 5-week EMST protocol (77) and 8-week EMST protocol (72) revealed no statistically significant differences in cough spirometry measures following training. Of high clinical relevance, however, was the observation that following 8-weeks of EMST set at 50% load, PEFR remained stable with 0% change despite the nature of this rapidly progressing motor neuron disease (72).

It should be appreciated, however, that certain EMST investigations while demonstrating improvements in MEP, may not be adequately powered to confirm changes in metrics of voluntary and reflex cough, may have inflated results due to small sample size, and/or may not directly test cough airflow. For example, Sasaki et al. (90), investigated the impact of EMST on MEP and PEFR in 33 healthy volunteers. Results revealed that compared to no-treatment controls, EMST in healthy subjects at both natural and fast expiratory flow rates increased MEP (*p* = 0.01), while no statistically significant improvements were observed in PEFR. Of clinical significance, however, EMST with natural expiratory flow rate increased PEFR from a mean of 8.4 (SD: 2.3) L/s to 8.9 (SD: 1.3) L/s post-training. Pilot investigations on EMST in multiple systems atrophy (89) have found direct improvements in MEPs, yet did not find statistically significant changes in PEFR. Similar limitations and/or findings have been observed in head and neck cancer patients (83, 87, 88).

In a retrospective case series involving head and neck cancer survivors experiencing chronic radiation-associated aspiration, investigators explored the therapeutic potential of EMST (83). Following an 8-week EMST program, MEPs significantly improved by an average of 57% (*p* < 0.001). This improvement in MEPs was associated with enhanced swallowing safety as per the Dynamic Imaging Grade of Swallowing Toxicity (DIGEST, *p* = 0.03); however, cough airflow outcomes were not directly measured in this study (83). Of interest, application of EMST in other populations with head and neck cancer have shown variable objective improvements in cough airflow. In six participants who underwent supracricoid partial laryngectomy for laryngeal cancer a mean increase in PEFR from 371.67 to 451.33 L/min or 21% was observed after 4-weeks of EMST (87). In contrast, in a small group of total laryngectomy participants (*n* = 10), PEFR did not improve after 4 and 8 weeks of EMST as compared to baseline (88). This may be due, in part, to altered physiologic anatomy and the inability to generate subglottic air pressure due to removal of the larynx—leaving these individuals dependent only on volume and speed of expiratory airflow to improve airway clearance (88). Despite these findings and limitations, these studies are essential as they highlight the need for larger, adequately powered investigations in these patient populations.

There is a paucity of data on the impact of IMST alone on objective cough outcomes. Comprehensive RST protocols which combine or assess both EMST and IMST have been investigated in stroke (86–89, 91, 92), ALS (76), Huntington's disease (78), and preoperative cardiac surgical patients (35). Kulnik et al. (82), aimed to examine the effect of expiratory muscle training, inspiratory muscle training, and a sham training protocol on cough airflow in acute stroke patients. Significant improvements in respiratory muscle strength (maximum inspiratory pressure, 14 cmH2O; *p* < 0.0001 and MEP, 15 cmH2O; *p* < 0.0001) were observed in all groups. Interestingly, voluntary cough (*p* = 0.0002) was significantly increased for the inspiratory and sham groups only. In combination with results from Hegland et al. (70), these two stroke investigations support the notion that training inspiratory muscles may be beneficial for improving voluntary cough effectiveness in this population, while training expiratory muscles may be beneficial for improving reflex cough airflow when implemented with a sufficient load (i.e., ≥60% of MEP) (70, 82). In alignment with these observations on resistive load, results from a prospective, single blinded, randomized controlled trial in unilateral stroke patients showed that 6-weeks of combined IMST and EMST at lower resistances (i.e., 30%–60% of maximum inspiratory pressure and 15%–75% of MEP) did not demonstrate statistically significant differences in PEFR as compared to standard of care control group. However, significant correlations were found between PEFR and MEP (r = 0.504, *p* < 0.05) (86). In a pilot investigation, a combined RST program in preoperative cardiac surgical patients found direct improvements in MEPs, yet did not find statistically significant changes in PEFR (35).

In a double-blind, randomized, sham-controlled trial with 45 individuals with early-stage ALS, significant gains in both MEPs and/or metrics of voluntary cough were observed after a 12-week combined inspiratory and expiratory RST program (76). Specifically, participants in the active RST group with 30% load, demonstrated significant increases in mean cough peak inspiratory flow. Voluntary cough peak inspiratory flow exhibited a significant difference between the active and sham RST groups. The group difference in mean cough peak inspiratory flow was 63.9 L/min (95% CI 17.3–110.5; *p* = 0.02). However, the change in cough PEFR did not reach statistical significance between the groups (mean difference = 59.4 L/min, 95% CI −15.4–134.2, *p* = 0.06). In another example, in an investigation on 18 individuals with Huntington's disease (78), a four-month home-based combined EMST and IMST program yielded significant improvements in several pulmonary measures including PEFR (*d* = 0.8) and MEP (*d* = 1.5). These findings are particularly relevant for neurodegenerative populations as resistance training for cough can be used as a proactive approach to address declining physiologic reserves and prolong expiratory pressure generation capacities essential for airway protection and defense (93).

### 2.4 Skill based rehabilitation of cough

A growing body of research supports the feasibility and effectiveness of task-specific, skill-based rehabilitation of cough. Cough skill training encompasses a range of rehabilitation treatment approaches which have been shown to improve metrics of voluntary and reflex cough through task-specific behavioral upregulation of cough (38, 85, 94–96). This has been achieved using both gold-standard spirometry (38, 94, 95), as well as with pragmatic tools primed for easy dissemination into clinical settings, including digital and analog peak flow meters (38, 85, 96). As there is a growing body of evidence on cough skill training, studies reviewed in this section will focus on randomized controlled trials, quasi-experimental investigations, cohort studies and single-subject experimental studies which report on objective cough outcome measures.

Early research in this area first demonstrated that cough can be volitionally upregulated in both healthy adults and individuals with Parkinson's disease (95, 97). Hegland and colleagues (97) investigated the extent to which 20 healthy, awake adults could voluntarily modify parameters of cough in response to 200 uM capsaicin, while recording cough airflow through spirometry and expiratory muscle activity via surface electromyography (sEMG). Results revealed that all participants were able to modify their reflex cough response to execute smaller or softer coughs and longer or louder coughs. Expanding upon this work, Brandimore et al. (95), investigated the voluntary upregulation of reflex cough in both healthy older adults and individuals with Parkinson's disease. Results revealed that healthy adults could enhance voluntary and reflex cough PEFR and cough expired volume through verbal instruction and visual biofeedback of airflow targets.

Importantly, these seminal studies substantiated that healthy adults can volitionally upregulate both reflex and voluntary cough function, and informed future investigations for individuals with Parkinson's disease. Thus, providing an essential basis for the development of behaviorally based treatments for hypotussic cough and informing future work targeting improvements in both reflex and voluntary cough function. Specifically, these studies (95, 97) informed the development of sensorimotor training for airway protection (smTAP) (38, 94, 95). smTAP is a distinct type of cough skill training that uses gold-standard spirometry for real-time cough airflow visualization and involves key features including: (a) a salient context for cough execution with a subthreshold level of cough-inducing capsaicin stimulus, (b) a verbal cue to “cough hard” for immediate enhancement of PEFR, (c) visual biofeedback of cough airflow for a target set at 25% above an individual's baseline cough PEFR, and (d) increased awareness of the urge-to-cough (29, 39, 98, 99) or cough sensations (38, 94, 95). Collectively, these parameters upregulate cough airflow comprehensively—targeting both sensory and motor components of cough. Importantly, smTAP recruits key principles of neuroplasticity (100) to promote the generalization of cough as a skilled sensorimotor behavior. Specifically, the goal of the treatment is to upregulate cough function in response to penetrant or aspirate material in the airway (10, 38, 94).

A recent prospective phase II randomized controlled trial compared the effects of smTAP and EMST in 65 individuals with Parkinson's disease (38). Results revealed that after 5-weeks of treatment, the smTAP group achieved improvements in both motor and sensory aspects of voluntary and reflex cough function. Following smTAP, voluntary cough PEFR improved by 0.51 L/s and reflex cough improved by 0.53 L/s. Comparatively, the EMST group demonstrated improved PEFR of 0.17 L/s and 0.23 L/s, respectively. Notably, the 18% improvement in mean voluntary PEFR for the smTAP group from 3.05 to 3.70 L/s is clinically relevant in light of recent findings that emphasize that cough strength of 3.41 L/s is effective for clearing ≥80% of subglottic aspirate material (21). This study represents the first randomized controlled trial to demonstrate that smTAP is effective in enhancing motor and sensory aspects of cough function, surpassing improvements seen in EMST, an established gold-standard treatment for airway protection in Parkinson's disease (38). Moreover, recent investigations in smTAP show that participants with Parkinson's disease demonstrate quick improvements during cough practice, suggesting rapid skill reacquisition, with unique trajectories of change over the course of the treatment (101).

Given that these findings suggest the strong potential benefit of smTAP to improve cough effectiveness, the same approach was trialed to determine feasibility in a group of fifteen individuals with PSP (94). After one session of smTAP, individuals with PSP demonstrated significant increases in PEFR (*p* < 0.001). Moreover, improvements in airflow variability for PEFR and cough expired volume were appreciated (*p* = 0.01). These findings demonstrate the immediate potential for individuals with PSP to enhance their cough function through smTAP and highlight the feasibility of cough rehabilitation in this population (94).

Voluntary cough skill training, in contrast to smTAP, occurs without a cough-inducing stimulus and may be completed either with gold standard spirometry or with a handheld peak flow meter. To date, four studies have examined voluntary cough skill training to rehabilitate cough dysfunction. Three of these studies have been completed in neurodegenerative diseases and one in healthy older adults (85, 95, 96, 102). In the first study to demonstrate that people with Parkinson's disease could volitionally upregulate voluntary cough effectiveness, investigators measured baseline voluntary sequential cough as well as voluntary cough modulation in 16 individuals with Parkinson's disease and 28 healthy age-matched controls (95). Participants were seated in front of a computer with direct visualization of cough airflow from spirometry and were provided with verbal instructions to exceed the target set at 25% above their maximum PEFR. Results showed that participants were able to increase PEFR and cough expired volume in the cueing condition for voluntary cough (*p* < 0.001) (95).

Recent data also highlights the feasibility of voluntary cough skill training conducted with widely available, low-cost clinical tools (96). In a single-subject treatment study, an 81-year-old individual with mid-stage Parkinson's disease participated in four voluntary cough skill training sessions. Sessions involved 16 sets of five voluntary cough trials utilizing a handheld analog peak flow meter. Each session was comprised of two parts: single coughs (Sets 1–8) and sequential coughs (Sets 9–16). Within these parts, four sets focused on practicing “strong” voluntary coughs (PEFR ≥250 L/min), while the remaining sets alternated between “medium-strong” (PEFR 150–249 L/min) and “weak” coughs (PEFR 60–149 L/min) to encourage varied practice. Results revealed that significant increases in both single voluntary cough PEFR (*p* = .008) and sequential voluntary cough PEFR (*p* = .029) were evident after voluntary cough skill training (96).

Further, a recent investigation demonstrated the practical feasibility of performing voluntary cough skill training with a handheld analog device via telehealth in 20 participants with neurodegenerative diseases including Parkinson's disease, Lewy body dementia, multiple system atrophy, and cerebellar ataxia (85). Participants completed four weeks of EMST and two weeks of cough skill training through clinician-directed telehealth sessions and home practice. In this investigations’ cough skill training protocol, voluntary single and sequential PEFR was collected at the start of each session with an analog peak flow meter. Sessions comprised of five sets of five coughs, including variation in cough types (single and sequential) and targets (25% above baseline for strong coughs and 25% below baseline for weak coughs) to enhance motor learning. Results revealed statistically significant increases in mean PEFR for single and sequential voluntary coughs from pre- to post-treatment (*p* < .001) (85).

In alignment with these results, findings from a single-blind, randomized controlled study in community-dwelling older adults revealed that a home-based, unsupervised four-week program using handheld peak flow meters showed an increase in voluntary PEFR compared to no-treatment controls (102). In contrast, individuals randomized to four-weeks of inspiratory muscle training demonstrated significant increases in maximum inspiratory pressures compared to cough training and control groups, but these effects did not translate to significant improvements in voluntary cough PEFR (102).

### 2.5 Compensatory interventions: internal vs. external techniques

Compensatory interventions focus on techniques that temporarily manage or minimize aspiration and are useful to maintain “safety” for a limited duration of time (103), without aiming for long-term restorative effects or addressing the underlying pathophysiology. Compensatory techniques aim to assist or augment cough in the short-term to minimize the depth and degree of airway invasion of aspirate material or to promote secretion mobilization. These techniques are often used in conditions where complete cough restoration is not possible or to support an individual through a specific phase of their condition. Alternatively, they may be used to minimize the impact of aspiration events while an individual is participating in a cough skill (59, 104–106) or strength-based rehabilitative treatment paradigm (103).

Internal strategies involve approaches that are largely driven by the individual to compensate for structural or physiologic impairments. Through an individual's own proprioceptive feedback and kinesthetic motor control (107), these approaches adapt levels of function to harness a transient improvement in cough and/or underlying mechanisms of airway protection (58, 59, 67, 103). Internal strategies are primarily volitional *maneuvers* which apply a cough or components of a cough in isolation or as part of a sequence of upper airway behaviors (105, 108, 109). Voluntary airway clearance maneuvers often require self-monitoring, the cognitive ability to retain multi-step instructions, and practice repeatedly. Further, these facilitative maneuvers require a relatively active level of participation by the individual performing them, as it is a strategy they must learn, apply, and generalize to the point of habituation during prandial behaviors (107).

In contrast, external strategies involve interventions that are exogenous to the individual and introduce an external action, manipulation or force to assist or augment cough production. This can occur *manually* with the help of another individual or item or *mechanically*, through the application of assistive devices, biomedical equipment or other technologies (56, 57, 110). These facilitative devices may modify, augment, assist or bypass structures which are not sufficient for effective cough generation (107). More frequently, external compensatory strategies minimize the need for high cognitive reserves and may be facilitated by or with a care provider (103).

### 2.6 Internal strategies for compensatory cough management

Speech-language pathologists commonly use internal compensatory cough strategies. Internal strategies for compensatory cough are at present, defined as airway maneuvers which incorporate a volitional or cued cough, or part of a cough, either independently or in a sequence. If confirmed with instrumental assessment, a cued voluntary cough after an aspiration event, may be effective to clear subglottic residue from the airway (21, 107). Alternatively, a volitional cough may be included as part of an airway protective sequence. For example, the supraglottic swallow and super-supraglottic swallow maneuver both incorporate a cough to clear residual food or liquid from the airway entrance and minimize risk for aspiration (109, 111, 112). Moreover, endoscopy can be used to provide feedback for the effectiveness of a maneuver or strategy. In a recent tutorial (105), a subjective review of endoscopic biofeedback targets for cough training with the application of compensatory strategies is reviewed.

### 2.7 External strategies for compensatory cough management

External compensatory cough management strategies are typically employed in consultation with other healthcare professionals such as respiratory therapy, physical therapy, pulmonology and neurology. External strategies for compensatory cough may be classified into proximal or peripheral airway clearance techniques (57). Proximal techniques aim to augment, support, or imitate cough by increasing PEFR with the goal of clearing mucous from the larger airways. This may be achieved by using devices or external pressure which assist inspiration (e.g., lung volume recruitment bag), assist expiration (e.g., manually assisted cough), or both (e.g., mechanical insufflation-exsufflation) (56, 57, 110, 113).

Proximal airway clearance techniques for assisted expiration help expiratory muscles to generate sufficient intra-abdominal and intrathoracic pressure necessary to increase the high velocity expiratory airflow produced during a cough (57). In contrast, those for assisted inspiration augment inspiratory lung volumes in single or stacked breaths (57). The application of these techniques inflate the respiratory system to the maximal desired volume, thereby increasing the inspiratory capacity which is associated with an increased PEFR (114). Assisted inspiration and expiration techniques combine an inflated respiratory system and increased inspiratory capacity with thoracic compression and increased expiratory airflow to optimize PEFR (57).

Peripheral airway clearance techniques are secretion mobilizing and aim to loosen secretions to improve ventilation (57, 115). Secretion mobilization is achieved by increasing expiratory flow above inspiratory, referred to as “bias expiratory flow”, to move secretions from the distal to proximal airways, where they can then be cleared with a cough or evacuated with respiratory equipment (52, 115). Peripheral airway clearance can be achieved through manual techniques, chest wall oscillation or compression, percussive ventilation and/or chest wall strapping (56, 57, 110, 113).

Although not exhaustive, Table 3 provides current examples of commonly used proximal and peripheral airway clearance techniques. These therapies are typically applied in conjunction with respiratory and pulmonology specialists but can complement the recommendations of SLPs for improved airway clearance (please see Supplementary File S1: Reading List). An excellent review by Chatwin et al. (57), highlights the specific indications and impact of each dedicated intervention on assisted and unassisted PEFR, as well as related pulmonary outcomes for respiratory and physical therapists.

**Table 3:** Airway clearance techniques.

Airway clearance technique	Classification (Proximal/Peripheral)	Type			Description	References
		Assisted inspiration	Assisted expiration	Secretion mobilization^a^		
Manually assisted cough (MAC)	Proximal		X		MAC uses a manual Heimlich, abdominal thrust maneuver, and/or costophrenic compression to increase PEFR by rapidly increasing intrathoracic pressure. This can be achieved with the assistance of another person or if the patient is mobile, self-applied through a stationary object such as a table.	(57, 116, 117)
Lung volume recruitment (LVR)	Proximal	X			Also known as “breath-stacking” or “air-stacking”, where multiple breaths are given by a device, such as an “ambu” style bag, manual resuscitator with or without a one-way valve, or positive pressure mouthpiece ventilation. Once inspiratory capacity is reached, the individual can actively or passively exhale or cough.	(57, 108, 110, 114, 117, 118)
Mechanical insufflation-exsufflation (MI-E)	Proximal	X	X		Also known as “cough assist machine”; MI-E delivers deep inspiration and expiration through a full face mask or artificial airway. It rapidly shifts from positive pressure (insufflation) to negative pressures (exsufflation) to simulate the airflow changes that occur during a cough, thereby increasing peak cough flow. This method is typically applied in patients where other strategies to improve peak expiratory flow rate are ineffective.	(57, 117, 119, 120)
High frequency chest wall oscillations or compressions	Peripheral			X	Compression or oscillation applied to the chest to move secretions to proximal airways for suction or cough. This can be achieved manually through hands/fingers or through a number of devices that envelop the chest such as an inflatable vest or jacket attached to an air pulse generator.	(57, 110, 115, 121)
Intrapulmonary percussive ventilation (IPV)	Peripheral			X	IPV refers to therapy delivered by a pneumatic device (e.g., intermittent positive pressure breathing machine) to deliver short, high frequency bursts of air and/or aerosol through a facemask, mouthpiece, or artificial airway to aid lung and airway clearance.	(57, 115, 121, 122)
Chest wall strapping (CWS)	Peripheral			X	CWS induces breathing at low lung volumes, increases elastic recoil and expiratory flow. Elastic material restricts the chest wall to passively lower functional residual capacity without use of expiratory muscles and may be beneficial for lung secretion clearance.	(57, 115, 123)

^a^ Loosening and mobilizing secretions from peripheral airways to central airways occurs with application of higher expiratory than inspiratory airflows or “bias expiratory flow”.

The complex management of respiratory dysfunction and the appropriate application of airway clearance techniques requires thoughtful discussion, unique patient-centric recommendations, and multidisciplinary assessment (124). Given the high variability and diverse indications and contraindications across various populations, both patients and care-providers may often be faced with a multitude of airway clearance options to select and manage, which can be a barrier to care. The Individual Management of Patient Airway Clearance (IMPACT) Program and advisory team has also developed an excellent multidisciplinary toolkit and virtual education platform to address the long-standing gap on education related to airway clearance options for individuals with chronic lung disease (56, 125). These resources are valuable and can help the clinical SLP gain familiarity with the number of evolving devices that can assist with airway clearance.

### 2.8 Practical implementation: intervention approaches for hypotussic cough

Here, we provide two practical case studies to demonstrate how to translate the current evidence on behavioral hypotussic cough management approaches to clinical practice. In the first clinical vignette, we highlight the importance and need for inter- and transdisciplinary collaboration. In the second, we emphasize how biobehavioral treatments of hypotussia may be implemented as a core component of a comprehensive aspiration management plan.

#### 2.8.1 Clinical case vignette 1

A 73-year-old female with spinal onset ALS presents to her routine multidisciplinary clinic appointment. She was diagnosed with ALS approximately one year ago by her local neurologist. She has since had a 30-pound weight loss and progressive decline in her respiratory status. Percutaneous endoscopic gastrostomy (PEG) and early noninvasive ventilation were previously declined. She has mild upper limb and severe lower limb impairment. She is largely dependent on her power-chair. Although she is able to self-feed, she reports that over the past six weeks she easily fatigues at mealtimes and requires increased assistance from family and/or funded in-home caretakers to finish meals. She reports increased difficulty managing her secretions and at least one choking episode on her saliva per night. The SLP uses a handheld peak flow meter to assess cough, her maximum PEFR out of six trials is 285 L/min. Based on pulmonary function testing completed with respiratory therapy, her forced vital capacity (FVC) is 2.40 L (82% predicted), maximum inspiratory pressure is 36 cmH2O (50% predicted) and MEP is 43 cmH2O (62% predicted). The SLP recommends a repeat videofluoroscopic exam given values which indicate increased risk for unsafe swallowing (PEFR <397 L/min) and ineffective airway clearance (PEFR <297 L/min) (21, 23). In a collaborative patient education session, the registered dietician and SLP provide care and support regarding her choice to decline PEG placement and remind her that they remain open to revisiting the conversation on PEG placement if her goals of care change over time (particularly in light of her FVC being above 50% to 60% predicted, a relevant factor for early PEG insertion and survival outcomes) in context of declining respiratory function (124, 126–128). Given that her FVC is ≥70% predicted, a mild-intensity 12-week combined RST program at 30% load is presented as a treatment option (76, 118, 128), to which she is receptive. Moreover, as her PEFR is greater than 270 L/min, the SLP recommends consultation with respiratory therapy for evaluation and consideration of proximal airway clearance techniques (i.e., manual assisted cough, lung volume recruitment, or “cough assist” mechanical insufflation-exsufflation techniques as clinically indicated) as proximal airway techniques may serve as a meaningful adjunct to augment airway protective behaviors and reduce risk of aspiration during meals (108, 118, 129). Cumulatively, the proactive combination of rehabilitative and compensatory approaches facilitated through an inter- and transdisciplinary care model, aim to improve airway protective behaviors, cough effectiveness, and avert the consequences of aspiration-related respiratory tract infections (93, 118, 124), while also leveraging joint decision making to align treatment goals with her choices and maintain patient autonomy.

#### 2.8.2 Clinical case vignette 2

A 68-year-old male with a five-year history of Parkinson's disease presents to an outpatient otolaryngology clinic on referral from his neurologist for increased difficulty swallowing. He presents with moderate rigidity and postural instability, festinating gait, hypokinetic dysarthria and sialorrhea. He finds that his chronic drooling is socially isolating and negatively impacts his quality of life. A clinical SLP and laryngologist collaborate to conduct a comprehensive assessment of airway protective behaviors. Cough assessment is performed with gold-standard cough spirometry. Maximum PEFR for single voluntary coughs is 2.2 L/s. In response to 200 uM of capsaicin, three reflex coughs are produced with a maximum PEFR of 1.2 L/s. Results from a respiratory manometer revealed a MEP of 59 cmH2O. Flexible endoscopic evaluation of swallowing is performed which reveals significant secretions pooling in the laryngopharynx at baseline. A standardized bolus protocol is completed and silent aspiration with thin liquids is observed and noted to increase in frequency and amount with increased volume. After each silent aspiration event, the clinicians ask the patient to rate his Urge to Cough (29, 98, 99) on a modified Borg scale, which he consistently rates as a two or three (out of ten). Cued voluntary coughs are ineffective at clearing ≥50% of subglottic residue, which aligns with clinically meaningful cutoffs for voluntary cough effectiveness in neurogenerative populations based on his PEFR (21). Prior to removing the endoscope, the clinicians ask the patient to visualize the screen. Endoscopic biofeedback on cough effectiveness in response to remaining subglottic aspirate is provided (105), which revealed stimulability for improving cough effectiveness with biofeedback and cueing. The patient is then provided with information for biobehavioral rehabilitation, which will target oral hygiene, secretion management, EMST and voluntary cough skill training. Specifically, the treatment plan for his first session will include calibration of his EMST device to 75% of MEP, setting his cough target to 25% above his maximum voluntary cough PEFR (i.e., 2.2 L/s to 2.75 L/s) on a handheld peak flow meter, and completing five sets of five repetitions on each device. In combination, targeting pulmonary function, coordination of cough, and secretion burden secondary to sialorrhea in therapy are expected to address patient-centric treatment targets. Moreover, this treatment paradigm may be engaging and feasible to complete at home via telehealth (85).

## 3 Future directions

Future research is needed in several areas to improve the management of hypotussic cough dysfunction. Although progress has been made as it pertains to observational and cross-sectional investigations which characterize hypotussia, there is a significant gap as it pertains to biobehavioral treatment studies. Primarily, large scale randomized controlled trials on the treatment of hypotussia in varied populations are needed. Moreover, longitudinal studies using gold-standard spirometry to quantify changes in cough function over time are required to more effectively screen, evaluate and treat hypotussic cough with a focus on long-term respiratory health outcomes. Additionally, the benefits of at-home handheld spirometry and pulmonary function tracking may enhance management of aspiration in these populations. Although clinical education efforts to promote interdisciplinary management of hypotussia are slowly emerging, accessible resources for continuing clinical education and best practice guidelines have yet to be established. Evaluation and treatment paradigms which introduce unique tussive agents (130, 131) or novel neurotherapeutics such as acute intermittent hypoxia (132–135) also require further investigation. Moreover, an enhanced understanding of how compensatory maneuvers and respiratory therapies impact objective measures of volitional cough effectiveness have yet to be fully elucidated (109).

## 4 Conclusions

In conclusion, hypotussia is a clinically relevant and viable treatment target for patients with dysphagia-mediated aspiration. The treatment of hypotussia may improve proximal airway clearance and minimize the negative sequelae associated with uncompensated, chronic aspiration. Treatments for hypotussia may include strength, skill or task-specific rehabilitation paradigms, complemented by adjuvant manual, mechanical or maneuver-based compensatory techniques. Moreover, there is sufficient evidence to suggest that the treatment of hypotussia in individuals with dysphagia is efficacious and practical to apply in real-world clinical settings. Members of an interdisciplinary aerodigestive team including neurologists, pulmonologists, sleep specialists, otolaryngologists, respiratory therapy, SLPs, and medical equipment specialists are all critical to providing patient centric care for individuals with hypotussia. Increasing the clinical uptake of specialized and interdisciplinary cough treatment paradigms may help improve patient health outcomes and quality of life.

## References

[1] Shen TY Pertzborn MC Rose MJ Musselwhite MN Davenport PW Bolser DC. Influence of intrathoracic vagotomy on the cough reflex in the anesthetized cat. Respir Physiol Neurobiol. (2022) 296:103805. 10.1016/j.resp.2021.103805 34678475 PMC8742786

[2] Lowell ER Borders JC Sevitz JS Dakin AE Brates D Troche MS. A primer on hypotussic cough: mechanisms and assessment. Curr Otorhinolaryngol Rep. (2023) 11(2):182–91. 10.1007/s40136-023-00446-5

[3] Palmer PM Padilla AH. Risk of an adverse event in individuals who aspirate: a review of current literature on host defenses and individual differences. Am J Speech Lang Pathol. (2022) 31(1):148–62. 10.1044/2021_AJSLP-20-00375 34731584

[4] Troche MS Brandimore AE Godoy J Hegland KW. A framework for understanding shared substrates of airway protection. J Appl Oral Sci. (2014) 22(4):251–60. 10.1590/1678-775720140132 25141195 PMC4126819

[5] Lee KK Davenport PW Smith JA Irwin RS McGarvey L Mazzone SB Global physiology and pathophysiology of cough: part 1: cough phenomenology - CHEST guideline and expert panel report. Chest. (2021) 159(1):282–93. 10.1016/j.chest.2020.08.2086 32888932 PMC8640837

[6] Kim JY Davenport PW Mou Y Hegland K. Primary site of constriction during the compression phase of cough in healthy young adults. Respir Physiol Neurobiol. (2023) 311:104033. 10.1016/j.resp.2023.104033 36764504 PMC10067529

[7] Silverman EP Hoffman-Ruddy B. Ch 10 respiration. In: Rousseau B, Branski RC, editors. Anatomy and Physiology of Speech and Hearing. New York, NY: Thieme Medical Publishers, Incorporated (2018). p. 311–50.

[8] Chung KF Widdicombe JG. Pharmacology and Therapeutics of Cough. Berlin: Springer-Verlag (2009). 18956491

[9] Spinou A. Non-pharmacological techniques for the extremes of the cough spectrum. Respir Physiol Neurobiol. (2018) 257:5–11. 10.1016/j.resp.2018.03.006 29530625

[10] Novaleski CK Near LA Benzo RP. Cough: an introductory guide for speech-language pathologists. Perspect ASHA SIGs. (2024) 9(1):75–91. 10.1044/2023_PERSP-23-00203

[11] Widdicombe J Singh V. Physiological and pathophysiological down-regulation of cough. Respir Physiol Neurobiol. (2006) 150(2–3):105–17. 10.1016/j.resp.2005.04.013 15878697

[12] McGarvey L Rubin BK Ebihara S Hegland K Rivet A Irwin RS Global physiology and pathophysiology of cough: part 2. Demographic and clinical considerations: cHEST expert panel report. Chest. (2021) 160(4):1413–23. 10.1016/j.chest.2021.04.039 33905678 PMC8692102

[13] Chung KF McGarvey L Song WJ Chang AB Lai K Canning BJ Cough hypersensitivity and chronic cough. Nat Rev Dis Primers. (2022) 8(1):45. 10.1038/s41572-022-00370-w 35773287 PMC9244241

[14] Sykes DL Morice AH. The cough reflex: the Janus of respiratory medicine. Front Physiol. (2021) 12:1005. 10.3389/fphys.2021.684080 PMC8277195 34267675

[15] Hegland K Sapienza C. SLP’s role in evaluation and treatment of cough function. Perspect Swal Swal Dis (Dysph). (2013) 22(3):85–93. 10.1044/sasd22.3.85

[16] Doruk C Curtis JA Dakin AE Troche MS. Cough and swallowing therapy and their effects on vocal fold bowing and laryngeal lesions. Laryngoscope. (2023) 134:1127–32. 10.1002/lary.30922 37497803

[17] Ebihara S Sekiya H Miyagi M Ebihara T Okazaki T. Dysphagia, dystussia, and aspiration pneumonia in elderly people. J Thorac Dis. (2016) 8(3):632–9. 10.21037/jtd.2016.02.60 27076964 PMC4805832

[18] Del Negro CA Funk GD Feldman JL. Breathing matters. Nat Rev Neurosci. (2018) 19(6):351–67. 10.1038/s41583-018-0003-6 29740175 PMC6636643

[19] Mandell LA Niederman MS. Aspiration pneumonia. N Engl J Med. (2019) 380(7):651–63. 10.1056/NEJMra1714562 30763196

[20] Bolser DC Davenport PW. Functional organization of the central cough generation mechanism. Pulm Pharmacol Ther. (2002) 15(3):221–5. 10.1006/pupt.2002.0361 12099768

[21] Borders JC Troche MS. Voluntary cough effectiveness and airway clearance in neurodegenerative disease. J Speech Lang Hear Res. (2022) 65(2):431–49. 10.1044/2021_JSLHR-21-00308 34936376

[22] Hegland KW Okun MS Troche MS. Sequential voluntary cough and aspiration or aspiration risk in Parkinson’s disease. Lung. (2014) 192(4):601–8. 10.1007/s00408-014-9584-7 24792231 PMC4740922

[23] Plowman EK Watts SA Robison R Tabor L Dion C Gaziano J Voluntary cough airflow differentiates safe versus unsafe swallowing in amyotrophic lateral sclerosis. Dysphagia. (2016) 31(3):383–90. 10.1007/s00455-015-9687-1 26803772 PMC4871758

[24] Prescott SL Liberles SD. Internal senses of the vagus nerve. Neuron. (2022) 110(4):579–99. 10.1016/j.neuron.2021.12.020 35051375 PMC8857038

[25] Lowell ER Borders JC Perry SE Dakin AE Sevitz JS Kuo SH Sensorimotor cough dysfunction in cerebellar ataxias. Cerebellum. (2023). 10.1007/s12311-023-01635-0. [Epub ahead of print]. 38032397 PMC11145628

[26] Foote AG Thibeault SL. Sensory innervation of the larynx and the search for mucosal mechanoreceptors. J Speech Lang Hear Res. (2021) 64(2):371–91. 10.1044/2020_JSLHR-20-00350 33465318 PMC8632506

[27] McGovern AE Mazzone SB. Neural regulation of inflammation in the airways and lungs. Auton Neurosci. (2014) 182:95–101. 10.1016/j.autneu.2013.12.008 24411267

[28] Bianchi AL Gestreau C. The brainstem respiratory network: an overview of a half century of research. Respir Physiol Neurobiol. (2009) 168(1):4–12. 10.1016/j.resp.2009.04.019 19406252

[29] Davenport PW. Clinical cough I: the urge-to-cough: a respiratory sensation. In: Chung KF Widdicombe J, editors. Pharmacology and Therapeutics of Cough. Berlin, Heidelberg: Springer (2009). p. 263–76. 10.1007/978-3-540-79842-2_13 18825345

[30] Lin TF Shune S. The mind–body–breath link during oral intake in chronic obstructive pulmonary disease: a grounded theory analysis. Dysphagia. (2022) 38:367–78. 10.1007/s00455-022-10473-x 35713729

[31] Mir MJ Childers J Wheeler-Hegland K. Cough correlates of functional swallow outcomes in atypical parkinsonism. Movement Disord Clin Pract. (2024) 11:265–75. 10.1002/mdc3.13965 PMC10928338 38229245

[32] Plowman-Prine EK Sapienza CM Okun MS Pollock SL Jacobson C Wu SS The relationship between quality of life and swallowing in Parkinson’s disease. Mov Disord. (2009) 24(9):1352–8. 10.1002/mds.22617 19425089 PMC3614344

[33] Ball L Meteyard L Powell RJ. Predictors of aspiration pneumonia: developing a new matrix for speech and language therapists. Eur Arch Otorhinolaryngol. (2023) 280(11):5101–14. 10.1007/s00405-023-08153-z 37543958

[34] Addington WR Stephens RE Gilliland KA. Assessing the laryngeal cough reflex and the risk of developing pneumonia after stroke: an interhospital comparison. Stroke. (1999) 30(6):1203–7. 10.1161/01.STR.30.6.1203 10356100

[35] Donohue C Wiele L Terry A Jeng E Beaver T Martin T Preoperative respiratory strength training is feasible, safe, and improves pulmonary physiologic capacity in individuals undergoing cardiovascular surgery. JTCVS Open. (2023) 15:324–31. 10.1016/j.xjon.2023.07.005 37808054 PMC10556933

[36] Marik PE Kaplan D. Aspiration pneumonia and dysphagia in the elderly. Chest. (2003) 124(1):328–36. 10.1378/chest.124.1.328 12853541

[37] Plowman EK Anderson A York JD DiBiase L Vasilopoulos T Arnaoutakis G Dysphagia after cardiac surgery: prevalence, risk factors, and associated outcomes. J Thorac Cardiovasc Surg. (2023) 165(2):737–46. 10.1016/j.jtcvs.2021.02.087 33814177

[38] Troche MS Curtis JA Sevitz JS Dakin AE Perry SE Borders JC Rehabilitating cough dysfunction in Parkinson’s disease: a randomized controlled trial. Mov Disord. (2023) 38(2):201–11. 10.1002/mds.29268 36345090

[39] Troche MS Brandimore AE Okun MS Davenport PW Hegland KW. Decreased cough sensitivity and aspiration in Parkinson disease. Chest. (2014) 146(5):1294–9. 10.1378/chest.14-0066 24968148 PMC4219343

[40] Hegland K W Troche MS Brandimore AE Davenport PW Okun MS. Comparison of voluntary and reflex cough effectiveness in Parkinson’s disease. Parkinsonism Relat Disord. (2014) 20(11):1226–30. 10.1016/j.parkreldis.2014.09.010 25246315 PMC5450039

[41] Tabor-Gray L Vasilopoulos T Wheeler-Hegland K Wymer J Plowman EK. Reflexive airway sensorimotor responses in individuals with amyotrophic lateral sclerosis. Dysphagia. (2021) 36(4):574–82. 10.1007/s00455-020-10171-6 32778945

[42] Tabor-Gray LC Gallestagui A Vasilopoulos T Plowman EK. Characteristics of impaired voluntary cough function in individuals with amyotrophic lateral sclerosis. Amyotroph Lateral Scler Frontotemporal Degener. (2019) 20(1–2):37–42. 10.1080/21678421.2018.1510011 30652513 PMC6513719

[43] Tabor-Gray L Vasilopoulos T Plowman EK. Differences in voluntary and reflexive cough strength in individuals with amyotrophic lateral sclerosis and healthy adults. Muscle Nerve. (2020) 62(5):597–600. 10.1002/mus.27040 32776561

[44] Chiara T Martin AD Davenport PW Bolser DC. Expiratory muscle strength training in persons with multiple sclerosis having mild to moderate disability: effect on maximal expiratory pressure, pulmonary function, and maximal voluntary cough. Arch Phys Med Rehabil. (2006) 87(4):468–73. 10.1016/j.apmr.2005.12.035 16571384 PMC3121162

[45] Borders JC Sevitz JS Curtis JA Vanegas-Arroyave N Troche MS. Sensorimotor cough dysfunction is prevalent and pervasive in progressive supranuclear palsy. Mov Disord. (2021) 36(11):2624–33. 10.1002/mds.28707 34173683

[46] Hammond CAS Goldstein LB Horner RD Ying J Gray L Gonzalez-Rothi L Predicting aspiration in patients with ischemic stroke: comparison of clinical signs and aerodynamic measures of voluntary cough. Chest. (2009) 135(3):769–77. 10.1378/chest.08-1122 19017886 PMC3121155

[47] Fullerton A Mou Y Silver N Chheda NN Hitchcock K Hegland K. Reflex vs. Volitional cough differences amongst head and neck cancer survivors characterized by time since treatment and aspiration status. Respir Physiol Neurobiol. (2021) 293:103702. 10.1016/j.resp.2021.103702 34033947

[48] Fullerton A Mou Y Silver N Chheda N Bolser DC Wheeler-Hegland K. Impact of tussigenic stimuli on perceived upper airway sensation and motor cough response following total laryngectomy. Front Physiol. (2020) 11:477. 10.3389/fphys.2020.00477 32547408 PMC7272598

[49] Hutcheson K Barrow MP Warneke CL Wang Y Eapen G Lai SY Cough strength and expiratory force in aspirating and nonaspirating postradiation head and neck cancer survivors: expiratory function and aspiration in HNC. Laryngoscope. (2018) 128(7):1615–21. 10.1002/lary.26986 29114887 PMC5940582

[50] Mishra A Malandraki GA Sheppard JJ Gordon AM Levy ES Troche MS. Voluntary cough and clinical swallow function in children with spastic cerebral palsy and healthy controls. Dysphagia. (2019) 34(2):145–54. 10.1007/s00455-018-9933-4 30088088

[51] Pitts L Hamilton VK Walaszek EA Watts S Cherney LR. Voluntary cough testing as a clinical indicator of airway protection in cervical spinal cord injury. Laryngoscope. (2023) 133(6):1434–41. 10.1002/lary.30369 36062957

[52] Dallal-York J Croft K Anderson A DiBiase L Donohue C Vasilopoulos T A prospective examination of swallow and cough dysfunction after lung transplantation. Neurogastroenterol Motil. (2023) 35(4):e14458. 10.1111/nmo.14458 36168190

[53] Mir MJ Hegland KW. A survey of speech-language pathologists’ experience with clinical cough assessment. Perspect ASHA SIGs. (2021) 6(6):1627–40. 10.1044/2021_PERSP-21-00144 PMC9084607 35546793

[54] American Speech Language Hearing Association. Adult Dysphagiaractice. American Speech-Language-Hearing Association (2023). (cited January 15, 2024). Available online at: https://www.asha.org/Practice-Portal/Clinical-Topics/Adult-Dysphagia/

[55] Swallowing and Swallowing Disorders (Dysphagia) (SIG13), American Board of Swallowing and Swallowing Disorders (AB-SSD) Joint Committee on Dysphagia Competencies. Dysphagia Competency Verification Tool (DCVT). American Board of Swallowing and Swallowing Disorders (2024) (cited January 15, 2024). Available online at: https://www.asha.org/siteassets/practice-portal/dysphagia-competency-verification-tool-users-guide.pdf

[56] Solomon GM Barker AF McSpiritt E Marikovics S Quittner AL. IMPACT BE advisory pilot program. Pilot evaluation of a management toolkit for airway clearance therapy in bronchiectasis (IMPACT BE). ATS Sch. (2023) 4(1):76–86. 10.34197/ats-scholar.2022-0061IN 37089683 PMC10117526

[57] Chatwin M Toussaint M Gonçalves MR Sheers N Mellies U Gonzales-Bermejo J Airway clearance techniques in neuromuscular disorders: a state of the art review. Respir Med. (2018) 136:98–110. 10.1016/j.rmed.2018.01.012 29501255

[58] Huckabee ML Mills M Flynn R Doeltgen S. The evolution of swallowing rehabilitation and emergence of biofeedback modalities. Curr Otorhinolaryngol Rep. (2023) 11(2):144–53. 10.1007/s40136-023-00451-8

[59] Zimmerman E Carnaby G Lazarus CL Malandraki GA. Motor learning, neuroplasticity, and strength and skill training: moving from compensation to retraining in behavioral management of dysphagia. Am J Speech Lang Pathol. (2020) 29(2S):1065–77. 10.1044/2019_AJSLP-19-00088 32650656

[60] Robbins J Butler SG Daniels SK Diez Gross R Langmore S Lazarus CL Swallowing and dysphagia rehabilitation: translating principles of neural plasticity into clinically oriented evidence. J Speech Lang Hear Res. (2008) 51(1):S276–300. 10.1044/1092-4388(2008/021) 18230851

[61] Clark HM. Neuromuscular treatments for speech and swallowing. Am J Speech Lang Pathol. (2003) 12(4):400–15. 10.1044/1058-0360(2003/086) 14658992

[62] Sapienza CM Ruddy BH Silverman EP Plowman EK. Ch. 54: expiratory muscle strength training as a therapy modality. In: Carrau RL, Murry T, Howell RJ, editors. Comprehensive Management of Swallowing Disorders. 2nd ed. San Diego, CA: Plural Publishing, Incorporated (2015). p. 509–25.

[63] Burkhead LM Sapienza CM Rosenbek JC. Strength-training exercise in dysphagia rehabilitation: principles, procedures, and directions for future research. Dysphagia. (2007) 22(3):251–65. 10.1007/s00455-006-9074-z 17457549

[64] Morgan LB. Exercise-Based dysphagia rehabilitation: past, present, and future. Perspect ASHA SIGs. (2017) 2(13):36–43. 10.1044/persp2.SIG13.36

[65] Ahmed S Martin AAD Smith BK. Inspiratory muscle training in patients with prolonged mechanical ventilation: narrative review. Cardiopulm Phys Ther J. (2019) 30(1):44–50. 10.1097/CPT.0000000000000092 31105474 PMC6521954

[66] Huff A Brown A Smith BK Pitts T. Mechanisms for successful rehabilitation of cough in Parkinson’s disease using expiratory muscle strength training. Perspect ASHA SIGs. (2017) 2(13):93–102. 10.1044/persp2.SIG13.93

[67] Huckabee ML Lamvik-Gozdzikowska K. Reconsidering rehabilitation for neurogenic dysphagia: strengthening skill in swallowing. Curr Phys Med Rehabil Rep. (2018) 6(3):186–91. 10.1007/s40141-018-0193-x

[68] Malandraki GA Hutcheson KA. Intensive therapies for dysphagia: implementation of the intensive dysphagia rehabilitation and the MD Anderson swallowing boot camp approaches. Perspect ASHA SIGs. (2018) 3(13):133–45. 10.1044/persp3.SIG13.133

[69] Novaleski CK Doty RL Nolden AA Wise PM Mainland JD Dalton PH. Examining the influence of chemosensation on laryngeal health and disorders. J Voice. (2023) 37(2):234–44. 10.1016/j.jvoice.2020.12.029 33455853 PMC8277875

[70] Hegland KW Davenport PW Brandimore AE Singletary FF Troche MS. Rehabilitation of swallowing and cough functions following stroke: an expiratory muscle strength training trial. Arch Phys Med Rehabil. (2016) 97(8):1345–51. 10.1016/j.apmr.2016.03.027 27130637

[71] Pitts T Bolser D Rosenbek J Troche M Okun MS Sapienza C. Impact of expiratory muscle strength training on voluntary cough and swallow function in Parkinson disease. Chest. (2009) 135(5):1301–8. 10.1378/chest.08-1389 19029430 PMC5931232

[72] Plowman EK Tabor-Gray L Rosado KM Vasilopoulos T Robison R Chapin JL Impact of expiratory strength training in amyotrophic lateral sclerosis: results of a randomized, sham-controlled trial. Muscle Nerve. (2019) 59(1):40–6. 10.1002/mus.26292 29981250

[73] Reyes A Castillo A Castillo J. Effects of expiratory muscle training and air stacking on peak cough flow in individuals with Parkinson’s disease. Lung. (2020) 198(1):207–11. 10.1007/s00408-019-00291-8 31720808

[74] Reyes A Castillo A Castillo J Cornejo I. The effects of respiratory muscle training on peak cough flow in patients with Parkinson’s disease: a randomized controlled study. Clin Rehabil. (2018) 32(10):1317–27. 10.1177/0269215518774832 29756459

[75] Troche MS Okun MS Rosenbek JC Musson N Fernandez HH Rodriguez R Aspiration and swallowing in Parkinson disease and rehabilitation with EMST: a randomized trial. Neurology. (2010) 75(21):1912–9. 10.1212/WNL.0b013e3181fef115 21098406 PMC2995389

[76] Plowman EK Gray LT Chapin J Anderson A Vasilopoulos T Gooch C Respiratory strength training in amyotrophic lateral sclerosis: a double-blind, randomized, multicenter, sham-controlled trial. Neurology. (2023) 100(15):e1634–42. 10.1212/WNL.0000000000206830 36805435 PMC10103108

[77] Plowman EK Watts SA Tabor L Robison R Gaziano J Domer AS Impact of expiratory strength training in amyotrophic lateral sclerosis: expiratory training in ALS. Muscle Nerve. (2016) 54(1):48–53. 10.1002/mus.24990 26599236 PMC4879103

[78] Reyes A Cruickshank T Nosaka K Ziman M. Respiratory muscle training on pulmonary and swallowing function in patients with Huntington’s disease: a pilot randomised controlled trial. Clin Rehabil. (2015) 29(10):961–73. 10.1177/0269215514564087 25552526

[79] Kim J Davenport P Sapienza C. Effect of expiratory muscle strength training on elderly cough function. Arch Gerontol Geriatr. (2009) 48(3):361–6. 10.1016/j.archger.2008.03.006 18457885

[80] Neves LF Reis MH Plentz RDM Matte DL Coronel CC Sbruzzi G. Expiratory and expiratory plus inspiratory muscle training improves respiratory muscle strength in subjects with COPD: systematic review. Respir Care. (2014) 59(9):1381–8. 10.4187/respcare.02793 24782553

[81] Martin AD Smith BK Davenport PD Harman E Gonzalez-Rothi RJ Baz M Inspiratory muscle strength training improves weaning outcome in failure to wean patients: a randomized trial. Crit Care. (2011) 15(2):R84. 10.1186/cc10081 21385346 PMC3219341

[82] Kulnik ST Birring SS Moxham J Rafferty GF Kalra L. Does respiratory muscle training improve cough flow in acute stroke? Pilot randomized controlled trial. Stroke. (2015) 46(2):447–53. 10.1161/STROKEAHA.114.007110 25503549

[83] Hutcheson KA Barrow MP Plowman EK Lai SY Fuller CD Barringer DA Expiratory muscle strength training for radiation-associated aspiration after head and neck cancer: a case series: eMST in HNC aspirators. Laryngoscope. (2018) 128(5):1044–51. 10.1002/lary.26845 28833185 PMC5823707

[84] Sapienza C Troche M Pitts T Davenport P. Respiratory strength training: concept and intervention outcomes. Semin Speech Lang. (2011) 32(1):21–30. 10.1055/s-0031-1271972 21491356

[85] Sevitz JS Borders JC Dakin AE Kiefer BR Alcalay RN Kuo SH Rehabilitation of airway protection in individuals with movement disorders: a telehealth feasibility study. Am J Speech Lang Pathol. (2022) 31(6):2741–58. 10.1044/2022_AJSLP-22-00063 36279509 PMC9911128

[86] Liaw MY Hsu CH Leong CP Liao CY Wang LY Lu CH Respiratory muscle training in stroke patients with respiratory muscle weakness, dysphagia, and dysarthria – a prospective randomized trial. Medicine (Baltimore). (2020) 99(10):e19337. 10.1097/MD.0000000000019337 32150072 PMC7478702

[87] Palmer AD Bolognone RK Thomsen S Britton D Schindler J Graville DJ. The safety and efficacy of expiratory muscle strength training for rehabilitation after supracricoid partial laryngectomy: a pilot investigation. Ann Otol Rhinol Laryngol. (2019) 128(3):169–76. 10.1177/0003489418812901 30463423

[88] Van Sluis KE Kornman AF Groen WG Van Den Brekel MWM Van Der Molen L Hoffman-Ruddy B Expiratory muscle strength training in patients after total laryngectomy; A feasibility pilot study. Ann Otol Rhinol Laryngol. (2020) 129(12):1186–94. 10.1177/0003489420931889 32527195

[89] Srp M Bartosova T Klempir J Lagnerova R Gal O Listvanova T Expiratory muscle strength training in multiple system atrophy: a pilot study. Movement Disord Clin Pract. (2023) 10(7):1060–5. 10.1002/mdc3.13765 PMC10354620 37476315

[90] Dakin AE Borders JC Curtis JA Hegland KW Troche MS. Maximal expiratory pressure and its link with cough airflow before and after expiratory muscle strength training in Parkinson's disease. Perspect ASHA SIGs. (2023):1–10. 10.1044/2023_PERSP-23-00065

[91] Sasaki M. The effect of expiratory muscle training on pulmonary function in normal subjects. J Phys Ther Sci. (2007) 19(3):197–203. 10.1589/jpts.19.197

[92] Kulnik ST Rafferty GF Birring SS Moxham J Kalra L. A pilot study of respiratory muscle training to improve cough effectiveness and reduce the incidence of pneumonia in acute stroke: study protocol for a randomized controlled trial. Trials. (2014) 15(1):123. 10.1186/1745-6215-15-123 24725276 PMC4021694

[93] Rogus-Pulia NM Plowman EK. Shifting tides toward a proactive patient-centered approach in dysphagia management of neurodegenerative disease. Am J Speech Lang Pathol. (2020) 29:1094–109. 10.1044/2020_AJSLP-19-00136 32650651 PMC7844336

[94] Borders JC Curtis JA Sevitz JS Vanegas-Arroyave N Troche MS. Immediate effects of sensorimotor training in airway protection (smTAP) on cough outcomes in progressive supranuclear palsy: a feasibility study. Dysphagia. (2022) 37(1):74–83. 10.1007/s00455-021-10251-1 33515312

[95] Brandimore AE Hegland KW Okun MS Davenport PW Troche MS. Voluntary upregulation of reflex cough is possible in healthy older adults and Parkinson’s disease. J Appl Physiol. (2017) 123(1):19–26. 10.1152/japplphysiol.00612.2016 28360120 PMC5538815

[96] Curtis JA Dakin AE Troche MS. Respiratory–swallow coordination training and voluntary cough skill training: a single-subject treatment study in a person with Parkinson’s disease. J Speech Lang Hear Res. (2020) 63(2):472–86. 10.1044/2019_JSLHR-19-00207 32078392

[97] Hegland KW Bolser DC Davenport PW. Volitional control of reflex cough. J Appl Physiol. (2012) 113(1):39–46. 10.1152/japplphysiol.01299.2011 22492938 PMC3774289

[98] Davenport PW. Urge-to-cough: what can it teach US about cough? Lung. (2008) 186(S1):107–11. 10.1007/s00408-007-9045-7 17952695

[99] Davenport PW Sapienza C Bolser DC. Psychophysical assessment of the urge-to-cough. Eur Respir J. (2002) 12:249–53.

[100] Kleim JA Jones TA. Principles of experience-dependent neural plasticity: implications for rehabilitation after brain damage. J Speech Lang Hear Res. (2008) 51(1):S225–39. 10.1044/1092-4388(2008/018) 18230848

[101] Borders JC Hegland KW Vanegas-Arroyave N Troche MS. Motor performance during sensorimotor training for airway protection in Parkinson’s disease. Am J Speech Lang Pathol. (2023) 32(6):2718–33. 10.1044/2023_AJSLP-23-00055 37668552

[102] Kaneko H Suzuki A Horie J. Effects of cough training and inspiratory muscle training on cough strength in older adults: a randomized controlled trial. Lung. (2022) 200(1):49–57. 10.1007/s00408-022-00509-2 35050397

[103] Gosa MM Dodrill P Robbins J. Frontline interventions: considerations for modifying fluids and foods for management of feeding and swallowing disorders across the life span. Am J Speech Lang Pathol. (2020) 29(2S):934–44. 10.1044/2020_AJSLP-19-00065 32650663 PMC7844338

[104] Chen WG Schloesser D Arensdorf AM Simmons JM Cui C Valentino R The emerging science of interoception: sensing, integrating, interpreting, and regulating signals within the self. Trends Neurosci. (2021) 44(1):3–16. 10.1016/j.tins.2020.10.007 33378655 PMC7780231

[105] Curtis JA. Endoscopic biofeedback training for cough and swallowing: the what, why, and how. Perspect ASHA SIGs. (2024):1–8. 10.1044/2023_PERSP-23-00190

[106] Dodderi T Muthukumar V Hedge PS Rai SPV Moolambally SR Balasubramanium RK A survey of speech-language pathologists’ applications of motor learning principles in dysphagia therapy in adults in India. J Speech Lang Hear Res. (2023) 66(10):3745–62. 10.1044/2023_JSLHR-23-00185 37672783

[107] Leonard R Larsen D. Chapter 10 the treatment plan. In: Leonard R, Kendall K, editors. Dysphagia Assessment and Treatment Planning: A Team Approach, Fourth Edition. San Diego, United States: Plural Publishing, Incorporated (2017). p. 169–220.Available online at: http://ebookcentral.proquest.com/lib/teacherscollege-ebooks/detail.action?docID=5509493 (cited January 15, 2024).

[108] Cleary S Misiaszek JE Wheeler S Kalra S Genuis SK Johnston WS. Lung volume recruitment improves volitional airway clearance in amyotrophic lateral sclerosis. Muscle and Nerve. (2021) 64(6):676–82. 10.1002/mus.27417 34505708 PMC9293446

[109] Wheeler-Hegland K Ashford J Frymark T McCabe D Mullen R Musson N Evidence-based systematic review: oropharyngeal dysphagia behavioral treatments. Part II–impact of dysphagia treatment on normal swallow function. J Rehabil Res Dev. (2009) 46(2):185–94. 10.1682/JRRD.2008.08.0094 19533532

[110] McHenry KL. Airway clearance strategies and secretion management in amyotrophic lateral sclerosis. Respir Care. (2023) 69:227–37. 10.4187/respcare.11215 PMC10898456 37816542

[111] Logemann JA. The Evaluation and Treatment of Swallowing Disorders. 2nd edn. Austin, TX: Pro-Ed Inc (1998). 10.1097/00020840-199812000-00008

[112] Logemann JA Pauloski BR Rademaker AW Colangelo LA. Super-supraglottic swallow in irradiated head and neck cancer patients. Head Neck. (1997) 19(6):535–40. 10.1002/(SICI)1097-0347(199709)19:6<535::AID-HED11>3.0.CO;2-4 9278762

[113] Chatwin M Simonds AK. Long-term mechanical insufflation-exsufflation cough assistance in neuromuscular disease: patterns of use and lessons for application. Respir Care. (2020) 65(2):135–43. 10.4187/respcare.06882 31690614

[114] Sheers NL O’Sullivan R Howard ME Berlowitz DJ. The role of lung volume recruitment therapy in neuromuscular disease: a narrative review. Front Rehabil Sci. (2023) 4:1164628. 10.3389/fresc.2023.1164628 37565183 PMC10410160

[115] Toussaint M Chatwin M Gonzales J Berlowitz DJ Toussaint M Chatwin M 228th ENMC international workshop: airway clearance techniques in neuromuscular disorders. Neuromuscul Disord. (2018) 28(3):289–98. 10.1016/j.nmd.2017.10.008 29395673

[116] Morrow B Argent A Zampoli M Human A Corten L Toussaint M. Cough augmentation techniques for people with chronic neuromuscular disorders. Cochrane Database Syst Rev. (2021) 4(4):CD013170. 10.1002/14651858.CD013170.pub2 33887060 PMC8092569

[117] Rose L Adhikari NK Leasa D Fergusson DA McKim D. Cough augmentation techniques for extubation or weaning critically ill patients from mechanical ventilation. Cochrane Database Syst Rev. (2017) 1(1). 10.1002/14651858.CD011833.pub2 28075489 PMC6353102

[118] Sales De Campos P Olsen WL Wymer JP Smith BK. Respiratory therapies for amyotrophic lateral sclerosis: a state of the art review. Chron Respir Dis. (2023) 20:147997312311759. 10.1177/14799731231175915 PMC10214054 37219417

[119] Chatwin M Wakeman RH. Mechanical insufflation-exsufflation: considerations for improving clinical practice. JCM. (2023) 12(7):2626. 10.3390/jcm12072626 37048708 PMC10095394

[120] Spinou A. A review on cough augmentation techniques: assisted inspiration, assisted expiration and their combination. Physiol Res. (2020) 69(Suppl. 1):S93–103. 10.33549/physiolres.934407 32228015 PMC8604061

[121] Gipsman AI Lapinel NC Mayer OH. Airway clearance in patients with neuromuscular disease. Paediatr Respir Rev. (2023) 47:33–40. 10.1016/j.prrv.2023.02.002 36894356 PMC10928549

[122] Reardon CC Christiansen D Barnett ED Cabral HJ. Intrapulmonary percussive ventilation vs incentive spirometry for children with neuromuscular disease. Arch Pediatr Adolesc Med. (2005) 159(6):526–31. 10.1001/archpedi.159.6.526 15939850

[123] Eberlein M Schmidt GA Brower RG. Chest wall strapping. An old physiology experiment with new relevance to small airways diseases. Annals ATS. (2014) 11(8):1258–66. 10.1513/AnnalsATS.201312-465OI PMC5469355 25172621

[124] Berlowitz DJ Mathers S Hutchinson K Hogden A Carey KA Graco M The complexity of multidisciplinary respiratory care in amyotrophic lateral sclerosis. Breathe. (2023) 19(3):220269. 10.1183/20734735.0269-2022 37830099 PMC10567075

[125] About IMPACT - IMPACT. (2020) (cited January 16, 2024). Available online at: https://impact-be.com/about-impact/

[126] Bond L Ganguly P Khamankar N Mallet N Bowen G Green B A comprehensive examination of percutaneous endoscopic gastrostomy and its association with amyotrophic lateral sclerosis patient outcomes. Brain Sci. (2019) 9(9):223. 10.3390/brainsci9090223 31487846 PMC6770872

[127] EFNS Task Force on Diagnosis and Management of Amyotrophic Lateral Sclerosis, Andersen PM Abrahams S Borasio GD de Carvalho M Chio A EFNS Guidelines on the clinical management of amyotrophic lateral sclerosis (MALS)–revised report of an EFNS task force. Eur J Neurol. (2012) 19(3):360–75. 10.1111/j.1468-1331.2011.03501.x 21914052

[128] Kao TH Perry BJ. The current state and future directions of swallowing care in amyotrophic lateral sclerosis. Curr Phys Med Rehabil Rep. (2023) 11(2):199–211. 10.1007/s40141-023-00396-5 PMC12352545 40821619

[129] Cleary S Misiaszek JE Kalra S Wheeler S Johnston W. The effects of lung volume recruitment on coughing and pulmonary function in patients with ALS. Amyotroph Lateral Scler Frontotemporal Degener. (2013) 14(2):111–5. 10.3109/17482968.2012.720262 22970725

[130] Lüthi-Müller E Kool J Mylius V Diesener P. A new therapeutic approach for dystussia and atussia in neurogenic dysphagia: effect of aerosolized capsaicin on peak cough flow. Dysphagia. (2022) 37:1814–21. 10.1007/s00455-022-10439-z 35430718 PMC9643184

[131] Wallace E Guiu Hernandez E Epton M Ploen L Huckabee ML Macrae P. A sensory stimulation protocol to modulate cough sensitivity: a randomized controlled trial safety study. Am J Speech Lang Pathol. (2020) 29(3):1423–33. 10.1044/2020_AJSLP-19-00180 32379483

[132] Sajjadi E Seven YB Ehrbar JG Wymer JP Mitchell GS Smith BK. Acute intermittent hypoxia and respiratory muscle recruitment in people with amyotrophic lateral sclerosis: a preliminary study. Exp Neurol. (2021) 347:113890. 10.1016/j.expneurol.2021.113890 34624328 PMC9488543

[133] Sutor T Cavka K Vose AK Welch JF Davenport P Fuller DD Single-session effects of acute intermittent hypoxia on breathing function after human spinal cord injury. Exp Neurol. (2021) 342:113735. 10.1016/j.expneurol.2021.113735 33951477 PMC8616729

[134] Welch JF Sutor TW Vose AK Perim RR Fox EJ Mitchell GS. Synergy between acute intermittent hypoxia and task-specific training. Exerc Sport Sci Rev. (2020) 48(3):125–32. 10.1249/JES.0000000000000222 32412926 PMC7416561

[135] Vose AK Welch JF Nair J Dale EA Fox EJ Muir GD Therapeutic acute intermittent hypoxia: a translational roadmap for spinal cord injury and neuromuscular disease. Exp Neurol. (2021) 347:113891. 10.1016/j.expneurol.2021.113891 34637802 PMC8820239

