# Supplementary material for: Hypotussic cough in persons with dysphagia: biobehavioral interventions and pathways to clinical implementation
Source: Front Rehabil Sci. 2024 Jun 12;5:1394110. doi: 10.3389/fresc.2024.1394110 (PMC11199739; doi:10.3389/fresc.2024.1394110)
Supplement: Supplementary file 1 [file Datasheet1.docx]

Supplementary File 1: Reading List

Hypotussic Cough in Persons with Dysphagia: Biobehavioral Interventions and Pathways to Clinical Implementation

Justine Dallal-York^1*^, Michelle S. Troche^1^

*** Correspondence:**Justine Dallal-York
jd3967@tc.columbia.edu

Chatwin, M., Toussaint, M., Gonçalves, M.R., Sheers, N., Mellies, U., Gonzales-Bermejo, J., Sancho, J., Fauroux, B., Andersen, T., Hov, B., Nygren-Bonnier, M., Lacombe, M., Pernet, K., Kampelmacher, M., Devaux, C., Kinnett, K., Sheehan, D., Rao, F., Villanova, M., Berlowitz, D., Morrow, B.M., 2018. Airway clearance techniques in neuromuscular disorders: A state of the art review. Respiratory Medicine 136, 98–110. https://doi.org/10.1016/j.rmed.2018.01.012

Huckabee, M.-L., Mills, M., Flynn, R., Doeltgen, S., 2023. The Evolution of Swallowing Rehabilitation and Emergence of Biofeedback Modalities. Curr Otorhinolaryngol Rep 11, 144–153. https://doi.org/10.1007/s40136-023-00451-8

Kleim, J.A., Jones, T.A., 2008. Principles of Experience-Dependent Neural Plasticity: Implications for Rehabilitation After Brain Damage. J Speech Lang Hear Res 51. https://doi.org/10.1044/1092-4388(2008/018)

Lee, K.K., Davenport, P.W., Smith, J.A., Irwin, R.S., McGarvey, L., Mazzone, S.B., Birring, S.S., CHEST Expert Cough Panel, 2021. Global Physiology and Pathophysiology of Cough: Part 1: Cough Phenomenology - CHEST Guideline and Expert Panel Report. Chest 159, 282–293. https://doi.org/10.1016/j.chest.2020.08.2086

Lowell, E.R., Borders, J.C., Sevitz, J.S., Dakin, A.E., Brates, D., Troche, M.S., 2023. A Primer on Hypotussic Cough: Mechanisms and Assessment. Curr Otorhinolaryngol Rep 11, 182–191. https://doi.org/10.1007/s40136-023-00446-5

McGarvey, L., Rubin, B.K., Ebihara, S., Hegland, K., Rivet, A., Irwin, R.S., Bolser, D.C., Chang, A.B., Gibson, P.G., Mazzone, S.B., Altman, K.W., Barker, A.F., Birring, S.S., Blackhall, F., Bolser, D.C., Braman, S.S., Brightling, C., Chang, A.B., Coté, A., Gibson, P., El Solh, A.A., Escalante, P., Field, S.K., Fisher, D., French, C.T., Grant, C., Harding, S.M., Harnden, A., Hill, A.T., Irwin, R.S., Kahrilas, P.J., Kavanagh, J., Keogh, K.A., Lane, A.P., Madison, J.M., Malesker, M.A., Mazzone, S., McGarvey, L., Murad, M.H., Narasimhan, M., Newcombe, P., Oppenheimer, J., Rubin, B., Russell, R.J., Ryu, J.H., Singh, S., Smith, M.P., Tarlo, S.M., Vertigan, A.E., 2021. Global Physiology and Pathophysiology of Cough: Part 2 Demographic and Clinical Considerations. Chest 160, 1413–1423. https://doi.org/10.1016/j.chest.2021.04.039

Novaleski, C.K., Near, L.A., Benzo, R.P., 2023. Cough: An Introductory Guide for Speech-Language Pathologists. Perspect ASHA SIGs 1–17. https://doi.org/10.1044/2023_PERSP-23-00203

Robbins, J., Butler, S.G., Daniels, S.K., Diez Gross, R., Langmore, S., Lazarus, C.L., Martin-Harris, B., McCabe, D., Musson, N., Rosenbek, J., 2008. Swallowing and Dysphagia Rehabilitation: Translating Principles of Neural Plasticity Into Clinically Oriented Evidence. J Speech Lang Hear Res 51. https://doi.org/10.1044/1092-4388(2008/021)

Zimmerman, E., Carnaby, G., Lazarus, C.L., Malandraki, G.A., 2020. Motor Learning, Neuroplasticity, and Strength and Skill Training: Moving From Compensation to Retraining in Behavioral Management of Dysphagia. Am J Speech Lang Pathol 29, 1065–1077. https://doi.org/10.1044/2019_AJSLP-19-00088
